# The Prevalence and Risk Factors of Residual Back Pain After Vertebroplasty for Osteoporotic Vertebral Compression Fractures: A Systematic Review and Meta‐Analysis

**DOI:** 10.1111/os.70095

**Published:** 2025-06-26

**Authors:** Wenlong Li, Bing Zhang, Chencheng Mei, Hui Li, Ruizheng Zhu, Hao Lin, Jianmin Wen, Yang Wu, Xianzhi Ma

**Affiliations:** ^1^ Beijing Hepingli Hospital Beijing People's Republic of China; ^2^ Beijing Huaxin Hospital (The First Hospital of Tsinghua University) Beijing People's Republic of China; ^3^ The Third Affiliated Hospital of Beijing University of Chinese Medicine Beijing People's Republic of China; ^4^ Wangjing Hospital China Academy of Chinese Medical Sciences Beijing People's Republic of China

**Keywords:** meta‐analysis, osteoporotic vertebral compression fractures, percutaneous vertebroplasty, residual back pain, systematic review

## Abstract

**Background:**

Osteoporotic vertebral compression fractures (OVCFs) are prevalent among the elderly. Percutaneous vertebroplasty (PVA) is a commonly adopted minimally invasive treatment, yet many patients endure residual back pain (RBP) posttreatment, affecting their recovery and quality of life. Given the inconsistent prevalence of RBP across studies and the multitude of influencing factors, a systematic review and meta‐analysis is necessary to determine its prevalence and identify risk factors.

**Methods:**

English (PubMed, Embase, Web of Science, Ovid, Cochrane Library) and Chinese (CNKI, WanFang Data, VIP, CBM) literature databases were systematically searched until December 31, 2023. A random‐effects meta‐analysis was used to pool prevalence rates from individual studies. The associations between the identified risk factors and RBP were also analyzed. Sensitivity and subgroup analyzes were performed to identify the source of heterogeneity and to compare the prevalence estimates across the groups. The Joanna Briggs Institute's (JBIs) quality assessment checklist was used to evaluate the quality of the included studies. The *I* ^2^ tests were used to assess heterogeneity among the studies.

**Results:**

A total of 5146 articles were collected. Finally, 26 articles involving 9703 participants were included. Among them, 1245 experienced RBP. The prevalence of RBP in individual studies ranged from 4.56% to 50.00%, with a median of 14.90%. The pooled prevalence was 16.3% (95% CI: 13.5%–19.1%). The prevalence was higher among females [16.1% (95% CI: 13.1%–19.1%)] than males [15.9% (95% CI: 12.5%–19.2%)]. Subgroup analysis based on evaluation time showed that the prevalence was higher at 3 months or more after surgery [total: 17.3% (95% CI: 13.2%–21.4%) vs. 15.7% (95% CI: 12.1%–19.2%), males: 16.5% (95% CI: 12.3%–20.6%) vs. 15.3% (95% CI: 11.0%–19.6%), females: 16.9% (95% CI: 12.6%–21.1%) vs. 15.5% (95% CI: 11.6%–19.5%)]. Regarding the risk factors, several factors demonstrated significant associations with RBP. Patients with low pre‐bone mineral density were more likely to experience RBP compared to those with higher density. Moreover, thoracolumbar fascia injury, unsatisfactory cement distribution, multiple vertebral fractures, and postoperative vertebral body height recovery rate were also identified as risk factors increasing the likelihood of RBP.

**Conclusion:**

RBP is common after PVA, indicating the imperative of intervention strategies to alleviate the suffering and reduce negative ramifications. Moreover, various risk factors should be comprehensively considered to accurately assess patients' conditions and formulate targeted treatment and rehabilitation plans to alleviate patients' RBP symptoms.

## Graphical abstract

A systematic review and meta‐analysis reveal that the overall prevalence of residual back pain after vertebroplasty for OVCFs is 16.3% (16.1% in females, 15.9% in males), with risk factors including thoracolumbar fascia injury, unsatisfactory cement distribution, low pre‐bone mineral density, multiple vertebral fractures, etc.

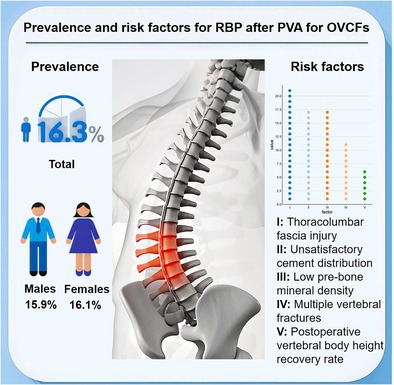

## 1 Introduction

Osteoporosis is a systemic bone metabolic disorder characterized by the decrease in bone mineral density and degradation of skeletal microarchitecture, resulting in increased bone fragility and risk of fracture [1, 2, 3, 4]. With the aging population, the incidence of osteoporosis and osteoporotic fractures has increased rapidly and become a global health issue [5, 6, 7]. China has the largest and fastest‐growing elderly population in the world, and studies have suggested that roughly 90 million elderly people (age ≥ 65 years) are living with osteoporosis [8, 9]. It is predicted that the population will increase to over 120 million by 2050 [10, 11]. Osteoporotic vertebral compression fractures (OVCFs) are one of the most common complications of osteoporosis. The fractures are becoming a common source of back pain and progressive spinal deformity, reducing quality of life and becoming an increasingly serious health problem [12, 13, 14]. Epidemiological studies have found that the incidence of vertebral fracture was 9.7% (95% CI, 8.2%–11.1%) among women and 10.5% (95% CI, 9.0%–12.0%) among men, respectively, at ages of 40 years or older [8].

At present, the main treatment options for OVCFs include conservative treatment and surgery. Percutaneous vertebroplasty (PVA) has become the most commonly used method because conservative treatment is difficult to relieve pain rapidly and restore vertebral stability in time [15, 16]. PVA is increasingly favored by orthopedists and patients because of its advantages of less trauma, rapid pain relief, better physiological function, and improved quality of life [17, 18]. However, in clinical practice, it is found that some patients experience residual back pain (RBP) in the original site after PVA. The reported prevalence of RBP after PVA varies across the studies from 4.56% to 23.74% [19, 20, 21, 22, 23], which not only makes patients suspect that the surgeons performed failed procedures, but also restricts clinical satisfaction.

No systematic review and meta‐analysis to date has estimated the consolidated prevalence and risk factors of RBP among elderly people after PVA. Evidence from such meta‐analysis will provide robust information on the epidemiology of RBP after PVA that would be necessary to plan early and suitable intervention strategies for those population groups. Therefore, the purpose of this review is to systematically analyze published studies on the prevalence and risk factors of RBP after PVA using both qualitative and quantitative methods.

## 2 Methods

We conducted the present study according to suggestions proposed by the Preferred Reporting Items for Systematic Reviews and Meta Analysis (PRISMA) Guidelines [24]. The protocol of this systematic review was registered in the international prospective register of systematic reviews (PROSPERO, registration number: CRD42024495236).

### 2.1 Literature Search Strategy

A literature search strategy was performed by two independent Researchers. English literature databases (PubMed, Embase, Web of Science, Ovid and Cochrane Library) and Chinese literature databases (CNKI, WanFang Data, VIP and CBM) were searched with publication dates from their inception to December 31, 2023. The Medical Subject Headings (MeSH) and non‐MeSH used in the present study included “Fractures, Compression,” “Spinal Fractures,” “Fractures, Bone,” “OVCF,” “vertebral fracture,” “osteoporotic compression fracture,” “compressed fracture,” “compression fracture,” “compressive fracture,” “Fracture, Compression,” “Compression Fractures,” “Fracture, Spinal,” “Fractures, Spinal,” “Spinal Fracture,” “Hangman Fracture,” “Fracture, Hangman,” “Hangman's Fracture,” “Fracture, Hangman's,” “Hangmans Fracture,” “Vertebroplasty,” “Kyphoplasty,” “PVA,” “percutaneous vertebral plasty,” “percutaneous kyphoplasty,” “Balloon Vertebroplasty,” “Vertebroplasty, Balloon,” “percutaneous vertebral augmentation,” “Pain, Postoperative,” “Postoperative Complications,” “Residual low back pain,” “Residual back pain,” “Residual pain,” “Postsurgical Pain,” “Postoperative Pain,” “Postoperative Pain, Chronic,” “Pain, Chronic Postsurgical,” “Chronic Postsurgical Pain,” “Postoperative Pain, Chronic,” “Acute Postoperative Pain,” “Postoperative Complication.”

### 2.2 Inclusion and Exclusion Criteria

Retrieved papers were included in this review if they satisfied the following criteria: (i) The study participants were elderly people (age ≥ 65 years) with OVCFs; (ii) Initial vertebroplasty treatment; (iii) The definition, evaluation time, and risk factors of RBP after vertebroplasty were clearly defined; (iv) reported the prevalence rates of RBP or reported the data for calculating the prevalence. Reviews, commentaries, case reports, and articles performed on animal subjects were excluded. Further, letters to the editor, conference papers, books, editorials, and notes were also excluded from the study.

### 2.3 Selecting Process

The selection process of included articles followed a consensus‐based approach. B Zhang and H Li reviewed all eligible articles separately, and the selection of included articles was based on their consensus. X Ma was invited for further consultation when there were divergences. Key design information, basic characteristics of the participants were extracted into a standardized evidence table, including first author(s)' name, sample size, year of publication, the country where the study was conducted, the number of positive cases, the corresponding prevalence estimates, the definition and evaluation time and risk factors of RBP after vertebroplasty. We also extracted the number of positive cases and the prevalence rates specifically for male and female participants.

### 2.4 Quality Assessment

The quality of all selected studies was assessed using the JBI Critical Appraisal Checklist [25]. The scoring of individual studies was conducted according to the frequency scales that were answered as yes, no, unclear, and not applicable.

### 2.5 Statistical Analysis

Statistical analyzes were performed using Stata 12.0 software (STATA Corporation, College Station, SE). Heterogeneity among studies was estimated using the Cochran Chi‐square test and the *I*‐square (*I* ^2^) test. When significant heterogeneity with *I* ^2^ ≥ 50% was observed, the random‐effects model analysis would be conducted. Otherwise, the fixed‐effect model method would be used. If there was statistical heterogeneity among the results, further sensitivity analysis would be performed to determine the source of heterogeneity. After excluding significant clinical heterogeneity, the randomized effect model method could be used for meta‐analysis. Because the prevalence of RBP after vertebroplasty treatment is only a descriptive result, not a differential comparison, there is usually no publication bias situation, so publication bias testing is not necessary in our study [26].

## 3 Results

### 3.1 Basic Characteristics of Included Studies

**FIGURE 1:**
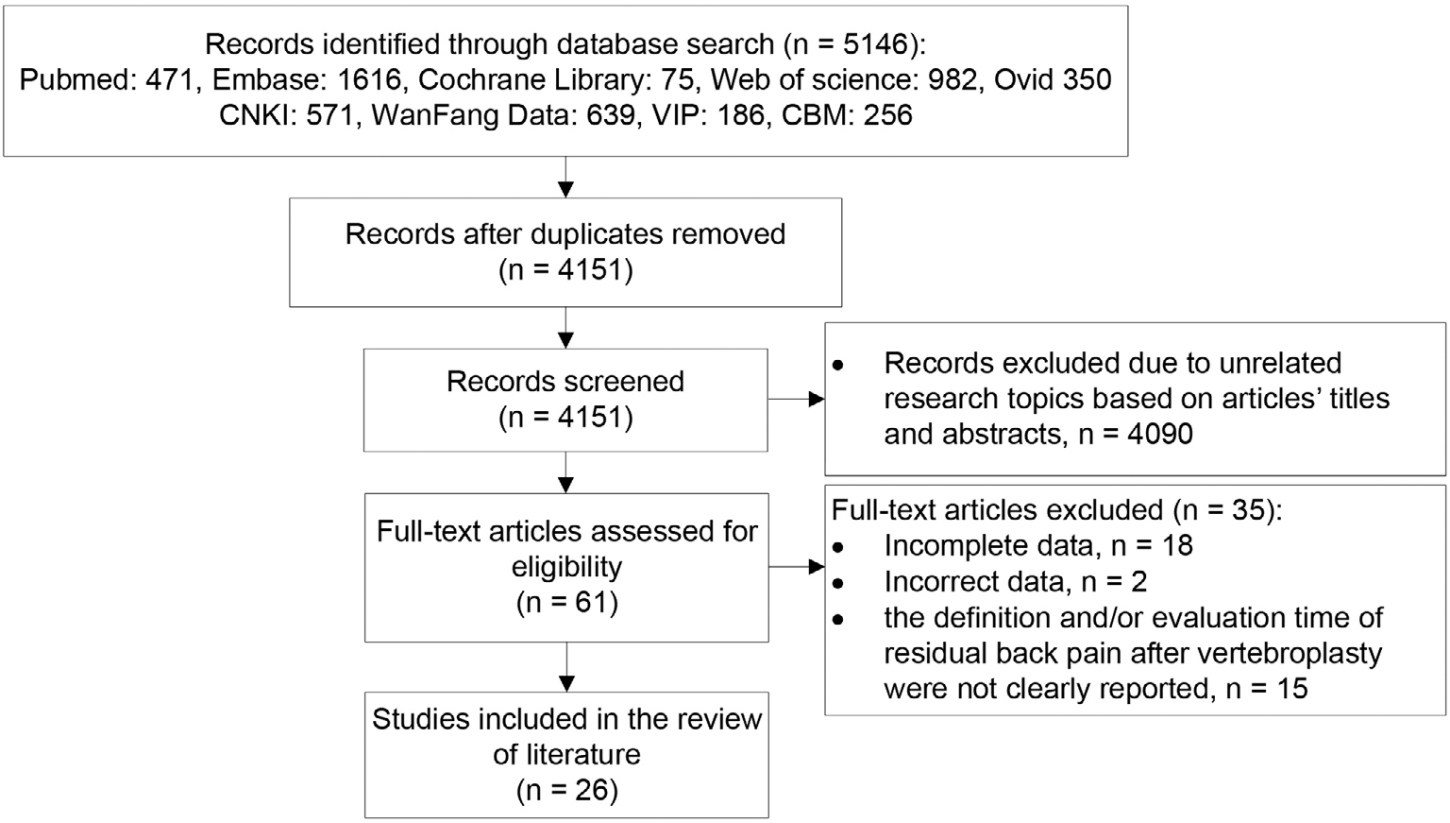
The article selection process.

A total of 5146 articles were collected. Of these, 995 were excluded due to duplication in data used, and 4090 were excluded due to unrelated research topics based on articles' titles and abstracts. Sixty‐one full‐text articles were then reviewed for eligibility, but 35 were excluded for the following reasons: incomplete data, incorrect data, the definition and/or evaluation time of RBP after vertebroplasty was not clearly reported. Finally, 26 articles [19, 20, 21, 22, 23, 27, 28, 29, 30, 31, 32, 33, 34, 35, 36, 37, 38, 39, 40, 41, 42, 43, 44, 45, 46, 47] were selected for the following meta‐analysis. The article selection process was illustrated in Figure 1.

All included 26 studies were published between 2016 and 2023, of which 25 were conducted in China, and one in Spain. A total of 9703 participants were included in this study, and samples for a single study range from 47 to 1316, with a median size of 250. Twenty studies [19, 20, 21, 23, 29, 30, 31, 32, 33, 34, 35, 36, 37, 38, 40, 41, 42, 43, 44, 45] defined RBP after PVA as postoperative VAS score greater than or equal to 4 points. In terms of evaluation time, 16 studies [19, 21, 27, 28, 31, 32, 33, 34, 36, 38, 39, 40, 42, 43, 44, 45] were evaluated within 3 months after surgery. The characteristics of the selected studies were summarized in Table 1.

**TABLE 1:** The characteristics of studies included in the systematic review and meta‐analysis.

Study ID	Country	Sample size			RBP after vertebroplasty		Number of RBP			Prevalence of RBP			Risk factors of RBP
		Total	Male	Female	Definition	Evaluation time	Total	Male	Female	Total	Male	Female	
Chang XB (2023) [27]	China	233	75	158	Compared with preoperative, the decrease of postoperative VAS score ≤ 5 points	One month after surgery	34	19	15	14.59%	25.33%	9.49%	I, II, III, IV, VI
Tian L (2023) [28]	China	228	94	134	Pain relief < 70%, pain relief = (preoperative VAS score‐postoperative VAS score)/preoperative VAS score	One month after surgery	35	16	19	15.35%	17.02%	14.18%	VII, XII, XVII
Su XH (2023) [29]	China	150	63	87	VAS score ≥ 4 points	Six months after surgery	30	14	16	20.00%	22.22%	18.39%	I, III, IV, V, XI
Liao BQ (2023) [30]	China	114	48	66	VAS score ≥ 4 points	Six months after surgery	41	19	22	35.96%	39.58%	33.33%	I, II, III, VIII
Li Y (2023) [31]	China	136	36	100	VAS score ≥ 4 points	One week after surgery	24	5	19	17.65%	13.89%	19.00%	I, II, III, VII
Cheng CG (2023) [32]	China	302	178	124	VAS score ≥ 4 points	One month after surgery	43	26	17	14.24%	14.61%	13.71%	I, II, VIII, XIII
Niu JJ (2023) [33]	China	221	40	181	VAS score ≥ 4 points	Three days after surgery	19	4	15	8.60%	10.00%	8.29%	I
Yu HW (2023) [22]	China	236	43	193	VAS score ≥ 3.5 points	Twelve months after surgery	30	4	26	12.71%	9.30%	13.47%	II, VI, VIII, X, XV
Wang ZW (2023) [34]	China	675	284	391	VAS score ≥ 4 points	One month after surgery	46	20	26	6.81%	7.04%	6.65%	I, II, III, IV, IX, X
Lin M (2023) [21]	China	281	49	232	VAS score ≥ 4 points	One day after surgery	47	3	44	16.73%	6.12%	18.97%	I, II, III, IX, XVIII
Gao X (2023) [35]	China	876	309	567	VAS score ≥ 4 points	Twelve months after surgery	86	34	52	9.82%	11.00%	9.17%	I, IX, XIII
Deng GH (2023) [20]	China	556	238	318	VAS score> 4 points	Six months after surgery	132	50	82	23.74%	21.01%	25.79%	III, IV, VII, XII, XIX
Wang R (2023) [23]	China	183	92	91	VAS score ≥ 4 points	Twelve months after surgery	28	15	13	15.30%	16.30%	14.29%	I, II, III, V, XI, XIV, XVI
Lin MM (2022) [36]	China	377	52	325	VAS score ≥ 4 points	One day after surgery	64	7	57	16.98%	13.46%	17.54%	I, II, III, IX, XVII
Liu C (2022) [37]	China	217	88	129	VAS score ≥ 4 points	Six months after surgery	33	13	20	15.21%	14.77%	15.50%	I, III, V, IV, XI
Chen C (2022) [38]	China	240	59	181	VAS score ≥ 4 points	One day after surgery	119	30	89	49.58%	50.85%	49.17%	I, III, V, VI, XXI
Yuan C (2021) [39]	China	112	64	48	VAS score ≥ 6 points	Two months after surgery	56	33	23	50.00%	51.56%	47.92%	I, II, III, IV, VI, X
Gao JF (2021) [40]	China	455	149	306	VAS score ≥ 4 points	One day after surgery	43	13	30	9.45%	8.72%	9.80%	I, II, III, IV, V, VII
Chen JG (2021) [41]	China	260	99	161	VAS score ≥ 4 points	Six months after surgery	40	15	25	15.38%	15.15%	15.53%	I, II, III, V, XI, XIV, XVI
Che XD (2021) [42]	China	150	62	88	VAS score> 4 points	One month after surgery	20	8	12	13.33%	12.90%	13.64%	I, II, III, IV, XII, XXII
Li Q (2021) [43]	China	268	53	215	VAS score ≥ 4 points	One month after surgery	37	7	30	13.81%	13.21%	13.95%	I, II, VIII, XIII
Liu C (2021) [44]	China	850	317	533	VAS score ≥ 4 points	One month after surgery	61	23	38	7.18%	7.26%	7.13%	I, II, III, IV, XII
Li Y (2020) [45]	China	809	/	/	Mean VAS score ≥ 4 points	1, 3, 7, 14 and 30days after the surgery	63	/	/	7.79%	/	/	I, II, VIII, IX
Yang JS (2019) [19]	China	1316	474	842	VAS score> 4 points	One month after surgery	60	19	41	4.56%	4.01%	4.87%	I, II, III, IV, VI, X
Huang TJ (2018) [46]	China	411	81	330	Compared with 1d before surgery, the decrease of three months after surgery VAS score ≤ 2 points	Three months after surgery	43	9	34	10.46%	11.11%	10.30%	VII
Peris P (2015) [47]	Spain	47	11	36	VAS score ≥ 7 points	Twelve months after surgery	11	2	9	23.40%	18.18%	25.00%	IV, XV, XX

*Note:* I, thoracolumbar fascia injury; II, unsatisfactory cement distribution; III, Low pre‐bone mineral density; IV, multiple vertebral fractures; V, postoperative vertebral body height recovery rate; VI, insufficient cement injected volume; VII, degree of vertebral compression; VIII, intravertebral vacuum cleft; IX, facet joint injury; X, depression; XI, body mass index; XII, bone cement leakage; XIII, paraspinal muscle fatty degeneration; XIV, recurrent fracture; XV, no antiosteoporotic treatment; XVI, nonunion; XVII, previous low back pain; XVIII, smoking; XIX, diabetes; XX, female; XXI, single or bilateral puncture; XXII, kümmel.

### 3.2 Quality of Included Studies

**FIGURE 2:**
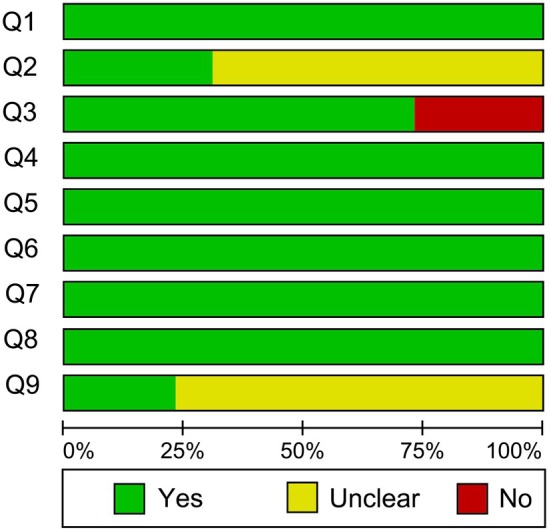
Quality of included studies.

**FIGURE 3:**
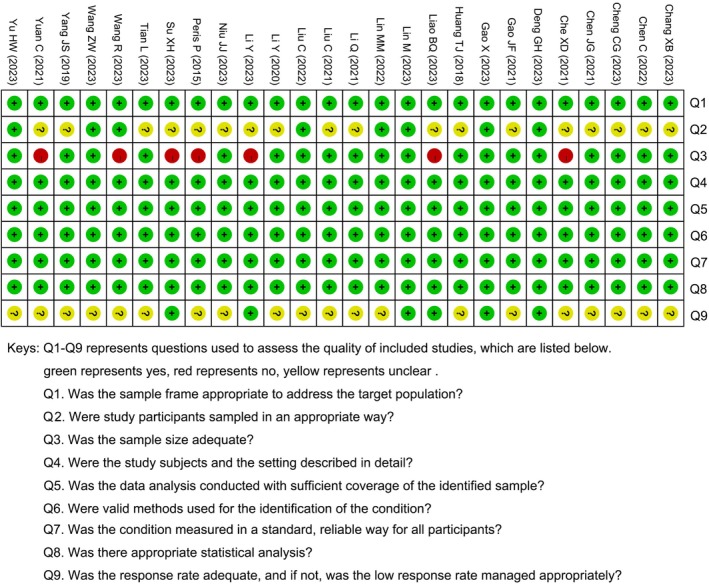
Quality of included studies summary (green represents yes, red represents no, yellow represents unclear).

Figures 2 and 3 show the quality and the risk of bias of studies included in this review. All included studies answered yes on Questions 1, 4, 5, 6, and 8. On Questions 2 and 9, a total of 18 [19, 27, 28, 29, 30, 31, 32, 33, 38, 39, 40, 41, 42, 43, 44, 45, 46, 47] and 20 [19, 22, 23, 27, 28, 32, 33, 34, 36, 37, 38, 39, 40, 41, 42, 43, 44, 45, 46, 47] studies answered unclear, respectively. For Questions 3, seven [23, 29, 30, 31, 39, 42, 47] studies answered no.

### 3.3 The Prevalence of RBP After PVA

**FIGURE 4:**
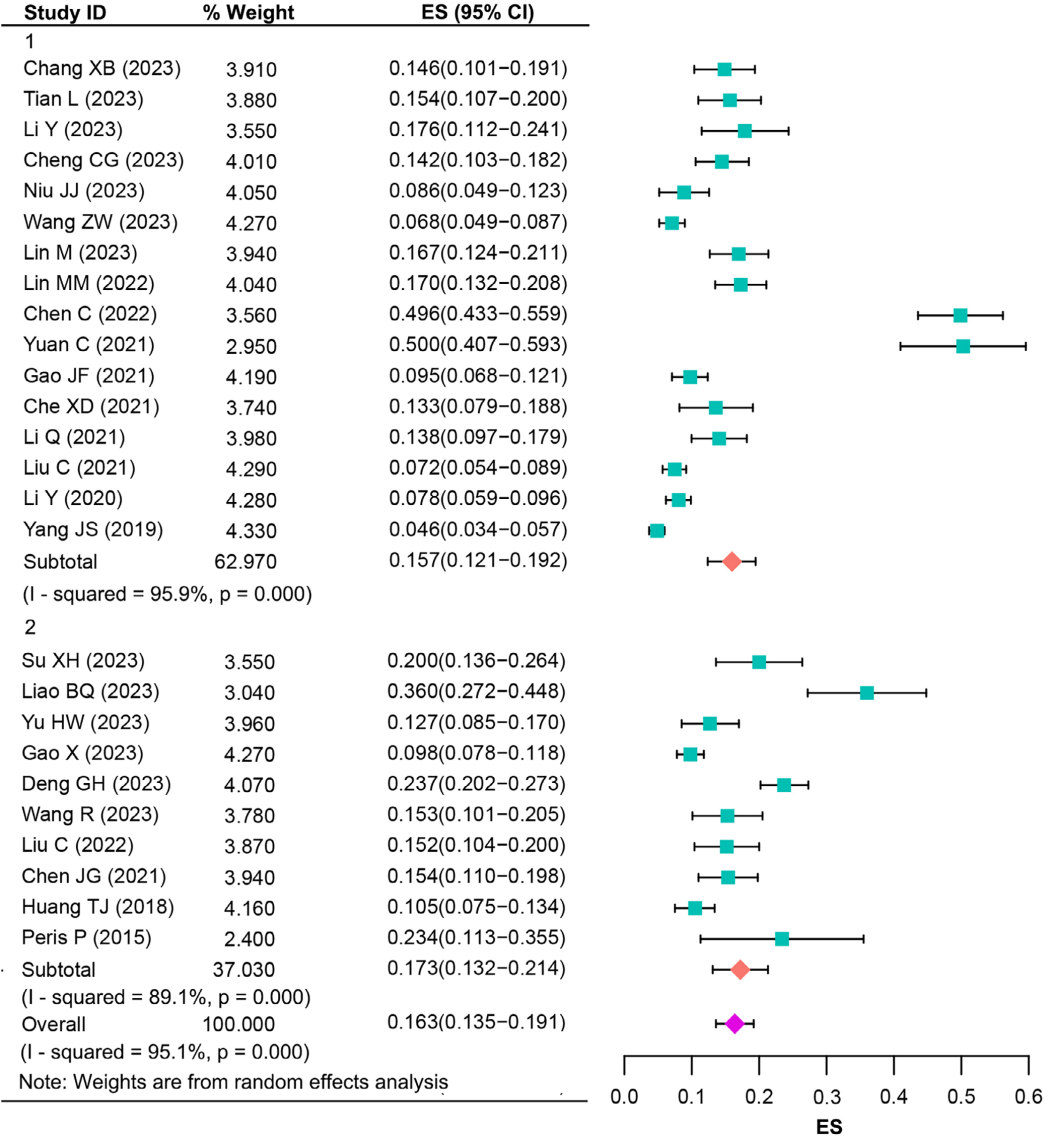
The prevalence of RBP after PVA.

Meta‐analysis was performed for the prevalence of RBP after PVA in all 26 studies [19, 20, 21, 22, 23, 27, 28, 29, 30, 31, 32, 33, 34, 35, 36, 37, 38, 39, 40, 41, 42, 43, 44, 45, 46, 47]. Of the 9703 participants, 1245 had experienced RBP. Prevalence of RBP for a single study ranged from 4.56% to 50.00%, with a median prevalence of 14.90%. Random‐effects model meta‐analysis indicated the pooled prevalence was found to be 16.3% (95% CI: 13.5%–19.1%). There was significant heterogeneity across the studies used for this analysis (*I* ^2^ = 95.1%, *p* < 0.001). In our subgroup analysis based on evaluation time, we found that the prevalence was higher for the studies whose evaluation time was 3 months or more after surgery [17.3% (*I* ^2^ = 89.1%, 95% CI: 13.2%–21.4%) vs. 15.7% (*I* ^2^ = 95.9%, 95% CI: 12.1%–19.2%), Figure 4].

**FIGURE 5:**
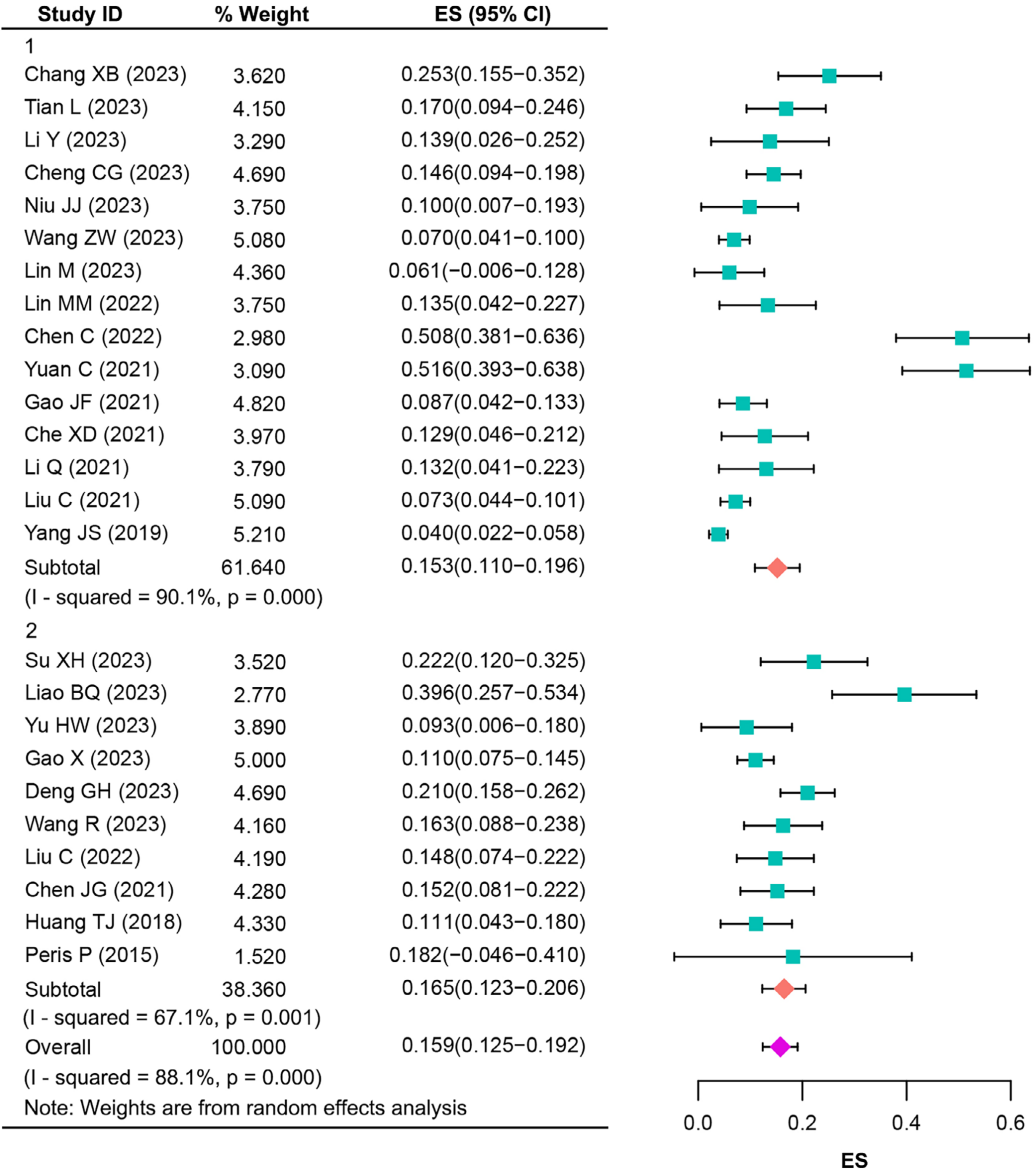
The prevalence of RBP after PVA in males.

**FIGURE 6:**
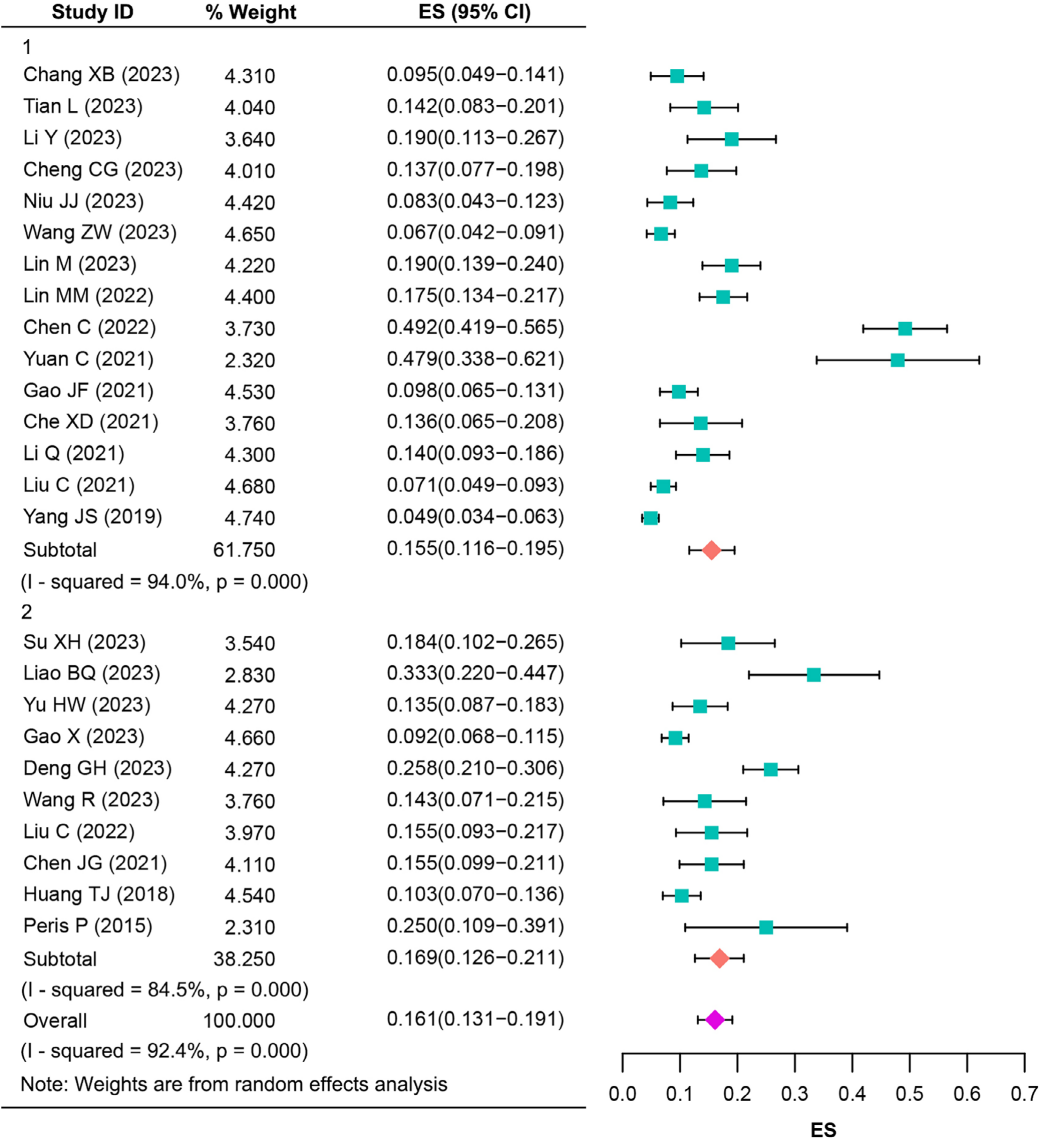
The prevalence of RBP after PVA in females.

Concerning the sex of the participants, we further explored the prevalence of RBP after PVA in different genders. Data were available for meta‐analysis in 25 studies [19, 20, 21, 22, 23, 27, 28, 29, 30, 31, 32, 33, 34, 35, 36, 37, 38, 39, 40, 41, 42, 43, 44, 46, 47]. Prevalence of RBP in males and females for a single study ranged from 4.01% to 51.56% and 4.87% to 49.17%, respectively, and there was a median prevalence of 13.89% and 14.18%. Random‐effects model meta‐analysis suggested a combined proportion of 15.9% (*I* ^2^ = 88.1%, 95% CI: 12.5%–19.2%) in males and 16.1% (*I* ^2^ = 92.4%, 95% CI: 13.1%–19.1%) in females, respectively. Subgroup analysis also observed higher prevalence for the studies whose evaluation time was 3 months or more after surgery in males [16.5% (*I* ^2^ = 67.1%, 95% CI: 12.3%–20.6%) vs. 15.3% (*I* ^2^ = 90.1%, 95% CI: 11.0%–19.6%), Figure 5] and females [16.9% (*I* ^2^ = 84.5%, 95% CI: 12.6%–21.1%) vs. 15.5% (*I* ^2^ = 94.0%, 95% CI: 11.6%–19.5%), Figure 6].

### 3.4 Sensitivity Analysis

**FIGURE 7:**
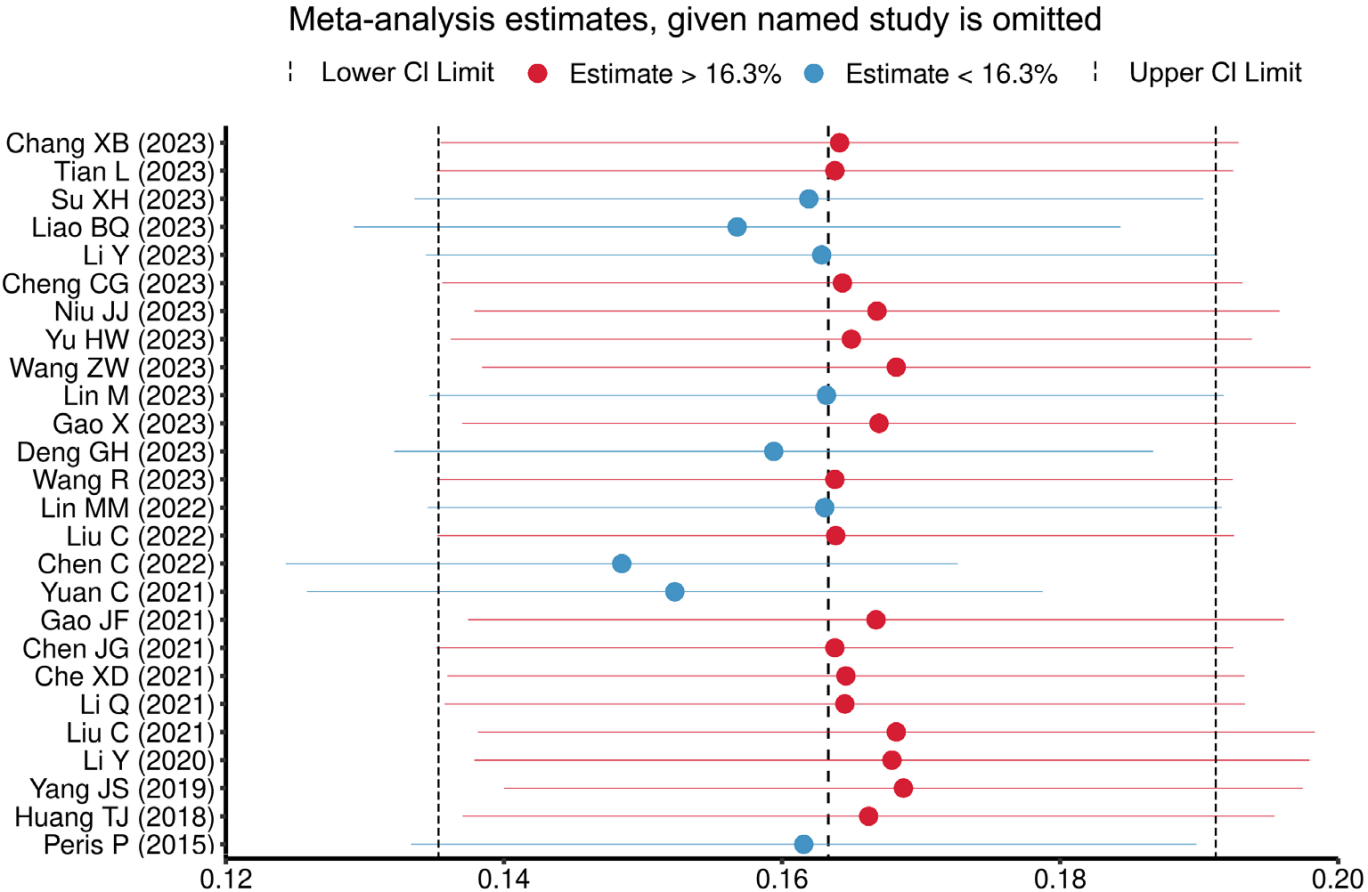
Sensitivity analysis.

We conducted a leave‐one‐out sensitivity analysis to further examine the possible cause of heterogeneity across the studies involved in the analysis. This analysis suggested that the findings of the main analysis are robust and not dependent on a single study. The pooled estimated prevalence of RBP after PVA varied between 14.8% (95% CI: 12.4%–17.3%) and 16.9% (95% CI: 14.0%–19.7%) after the deletion of a single study (Figure 7).

### 3.5 The Risk Factors of RBP After PVA

**FIGURE 8:**
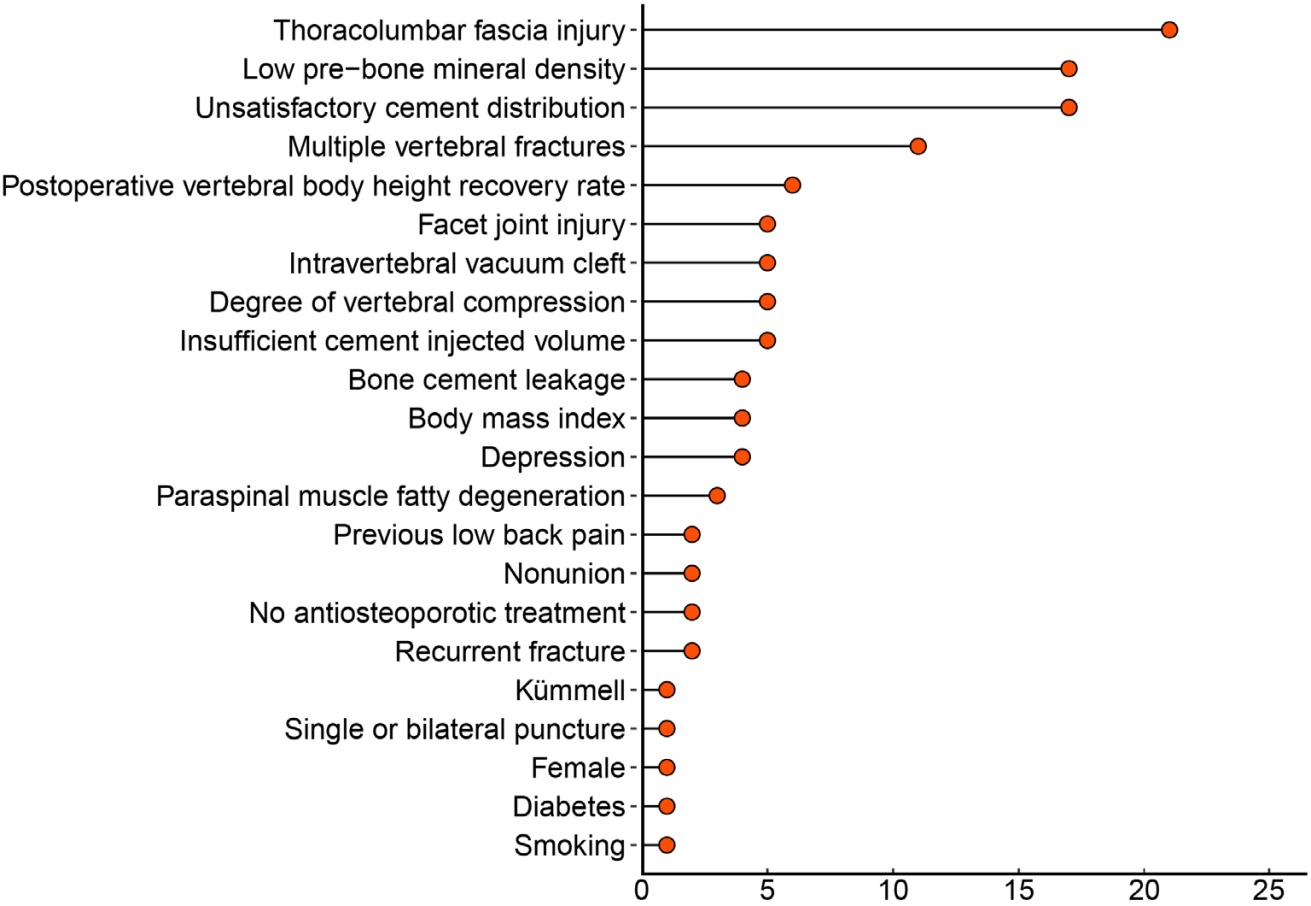
The risk factors of RBP after PVA.

In terms of risk factors related to RBP after PVA, we found that the top 5 factors were thoracolumbar fascia injury, unsatisfactory cement distribution, low pre‐bone mineral density, multiple vertebral fractures, and postoperative vertebral body height recovery rate. These factors were mentioned in 21 [19, 21, 23, 27, 28, 29, 30, 32, 33, 34, 35, 36, 37, 38, 39, 40, 41, 42, 43, 44, 45],17 [19, 21, 22, 23, 27, 30, 31, 32, 34, 36, 39, 40, 41, 42, 43, 44, 45],17 [19, 20, 21, 23, 27, 29, 30, 31, 34, 36, 37, 38, 39, 40, 41, 42, 44],11 [19, 20, 27, 29, 34, 37, 39, 40, 42, 44, 47] and 6 [23, 29, 37, 38, 40, 41] studies respectively. Five studies reported insufficient cement injected volume [19, 22, 27, 38, 39]. Five studies also reported the degree of vertebral compression [20, 28, 31, 40, 46]. There were five studies reporting intravertebral vacuum cleft [22, 30, 32, 43, 45]. Likewise, five studies reported facet joint injury [21, 34, 35, 36, 45]. Furthermore, it was found that depression was reported in 4 studies [19, 22, 34, 39]. Similarly, body mass index was reported in 4 studies [23, 29, 37, 41]. In addition, bone cement leakage was reported in 4 studies [20, 28, 42, 44]. Moreover, paraspinal muscle fatty degeneration was reported in 3 studies [32, 35, 43]. Recurrent fracture was reported in 2 studies [23, 41]. Additionally, no antiosteoporotic treatment was reported in 2 studies [22, 47]. Nonunion was also reported in 2 studies [23, 41]. Previous low back pain was reported in 2 studies [28, 36]. Smoking was reported in 1 study [21]. Diabetes was reported in 1 study [20]. Female was reported in 1 study [47]. Single or bilateral puncture was reported in 1 study [38], and kümmell was reported in 1 study [42]. (Figure 8).

## 4 Discussion

### 4.1 Systematic Analysis of RBP Prevalence and Risk Factors

Although PVA is currently accepted as a satisfactory and minimally invasive surgery for the treatment of OVCFs, which can rapidly relieve pain, restore patients' mobility early, and improve their quality of life, RBP after PVA is not rare and can be clinically intractable [48, 49]. The aim of the current study was to systematically analyze the prevalence and risk factors of RBP after PVA. The main findings of the current study were summarized as follows: our meta‐analysis showed that 16.3% of patients experienced RBP after PVA, which was 15.9% and 16.1% in males and females, respectively. Further systematic review supported that the prevalence was higher at 3 months or more after surgery. In terms of risk factors, we found that the top 5 factors were thoracolumbar fascia injury, unsatisfactory cement distribution, low pre‐bone mineral density, multiple vertebral fractures, and postoperative vertebral body height recovery rate. Therefore, early identification of patients with RBP after PVA is of great importance and may aid in delivering proper treatment and optimizing the use of resources.

### 4.2 Heterogeneity in RBP Prevalence and Evaluation Criteria

Our qualitative and quantitative synthesis indicated that the existing scientific evidence on the prevalence of RBP after PVA demonstrated a considerable variation depending on the country and type of study published, the gender of the participants, as well as the reported quality of the studies, ranging from 4.56% [19] to 50.00% [39]. One of the possible reasons for this variation could be differences in the definition and evaluation time. In our Meta‐Analysis, most studies (20/26) defined a VAS score greater than or equal to 4 points as a cutoff to distinguish whether pain was unsatisfactorily relieved. According to the VAS score system [50, 51, 52], a pain of VAS ≥ 4 is classified as moderate pain that can affect sleep, leading to impaired quality of life, and can usually be controlled with oral analgesics. We consider this threshold reasonable, as it aligns with clinical classifications of moderate pain that necessitate intervention and reflects the functional impact of persistent pain on patients' daily activities. The consistency of this criterion across the majority of included studies further supports its utility in defining RBP after PVA, balancing sensitivity to detect meaningful pain with specificity to avoid overdiagnosis of trivial discomfort.

In terms of evaluation time, there are significant differences between studies. In this study, we statistically analyzed the incidence of RBP after PVA at different evaluation times using 3 months postoperatively as the boundary. This temporal cutoff was selected based on the key consideration that 3 months represents a clinically meaningful threshold to distinguish acute postoperative pain from persistent pain syndromes. The results showed that the prevalence was higher at 3 months or more after surgery, which suggests RBP is an important public health issue requiring urgent attention in terms of early prevention and treatments. We therefore propose that 3 months postPVA serve as a standardized benchmark for evaluating RBP in both clinical practice and research, as it effectively differentiates transient postsurgical discomfort from clinically relevant persistent pain, thereby enabling targeted interventions to address underlying chronic mechanisms and improve long‐term outcomes. The included studies were highly geographically concentrated, with 25/26 (96.2%) originating from Chinese clinical settings and only one from Spain. Sensitivity analyzes excluding the sole non‐Chinese study still yielded a stable pooled prevalence of RBP: 16.2% (95% CI: 13.3%–19.0%). This could introduce regional bias. This concentration likely reflects China's unique epidemiological profile: China possesses the largest and most rapidly expanding elderly population globally. Research indicates that approximately 90 million individuals aged 65 years and older are currently affected by osteoporosis [8, 9]. Projections suggest that this demographic will surpass 120 million by the year 2050 [10, 11]. These disparities highlight the need for multinational studies to validate RBP prevalence in populations with differing healthcare resource allocations and treatment protocols.

### 4.3 Key Risk Factors and Pathophysiological Mechanisms

#### 4.3.1 Thoracolumbar Fascia Injury

A few studies have elaborated on the possible risk factors for RBP after PVA [20, 21, 22, 23, 34, 35]. In our Meta‐Analysis, thoracolumbar fascia injury ranked first. There is a close relationship between thoracolumbar fascia injury and RBP after PVA [53, 54, 55]. Multiple factors, such as surgical procedures, biomechanical changes, and nerve conduction mechanisms, may work together to lead to the emergence of RBP [56, 57]. During the performance of PVA, procedures such as puncture may indirectly damage the thoracolumbar fascia. The fascia tissues around the path traversed by the puncture needle may endure mechanical injuries like traction and tearing, destroying their original structural integrity. Furthermore, the local inflammatory response after the operation may also affect the thoracolumbar fascia, resulting in conditions such as hyperemia and edema in the fascia, which will then influence its normal functions and trigger pain [58, 59]. In terms of biomechanics, although the strength of the vertebra has been improved to some extent after PVA, the biomechanical balance of the entire spine is still in a stage of readjustment [57]. As the thoracolumbar fascia is an important structure involved in maintaining spinal stability, when it is damaged due to surgery or other related factors, it cannot well coordinate with other structures such as muscles to cope with this biomechanical change, resulting in unbalanced force on the lumbar muscles and affected spinal stability, which easily leads to persistent pain in the lumbar back and becomes an important predisposing factor for RBP [60, 61, 62, 63]. There is a rich distribution of nerve endings in the thoracolumbar fascia [64, 65, 66]. When it is damaged due to surgery or other reasons, these nerve endings will be stimulated and transmit pain signals to the central nervous system through the nerve conduction pathways, making patients feel pain in the lumbar and back regions. Moreover, there may be an accumulation of some pain‐causing substances around the damaged thoracolumbar fascia, which further sensitizes the nerve endings and aggravates the pain sensation [67, 68]. Even if the vertebra itself has undergone reinforcement, there will still be this kind of RBP caused by fascia damage.

#### 4.3.2 Unsatisfactory Cement Distribution

Unsatisfactory cement distribution is another important factor causing RBP [19, 45, 69]. If the bone cement is unevenly distributed within the vertebrae, it will lead to an unbalanced force on different parts of the vertebral [70, 71, 72]. Some areas will bear excessive pressure while the adjacent areas are relatively underloaded, which disrupts the original biomechanical balance of the entire spine. In order to adapt to this abnormal stress state, the surrounding structures such as muscles and ligaments need to make additional compensatory efforts. It is prone to cause muscle fatigue and strain, and then result in low back pain, which has become an important reason for RBP [19].

#### 4.3.3 Low Pre‐Bone Mineral Density

For patients with low pre‐bone mineral density, factors such as poor bone quality, problems with the bonding of bone cement, and an increased risk of re‐fracture work together, which may result in the occurrence and persistent existence of RBP [73, 74]. Although the vertebra has been strengthened to a certain extent through PVA, the improvement in the overall bone strength is still limited. During daily activities, the spine needs to bear pressure or torsions from different directions of the body. Compared with the vertebra with normal bone density, the vertebra with low bone density is more prone to have micro‐fractures and deformations [75, 76], which will then continuously stimulate the surrounding nerves, muscles and ligaments, and other tissues, triggering low back pain and becoming an inherent hidden danger for the occurrence of RBP. Due to low bone density, problems like poor adhesion between the bone cement and the vertebral body may occur after the bone cement is injected [23, 77]. During subsequent activities, the bone cement is prone to loosening and displacement, which will undermine the stability within the vertebral body and that of the whole spine, resulting in an unbalanced force on the spine. This makes the structures such as the muscles around the waist need to continuously compensate. Over time, it is easy to cause muscle fatigue and strain, thereby inducing RBP. Low pre‐bone mineral density significantly increases the risk of refracture of the vertebra after PVA [78, 79]. Once a refracture occurs, it will lead to the exacerbation of the already existing low back pain or the recurrence of the pain that has been alleviated, becoming an important factor for the persistent existence or even aggravation of RBP.

#### 4.3.4 Multiple Vertebral Fractures and Postoperative Vertebral Height Restoration Rate

Multiple vertebral fractures and postoperative vertebral body height recovery rate are also closely related to RBP after PVA [19, 80]. When multiple vertebral fractures occur, the integrity of multiple vertebrae is damaged, and even after vertebral augmentation, it is difficult to completely restore the overall biomechanical balance of the spine to its original state [34, 47]. If the postoperative vertebral height restoration rate is low, it means that the vertebrae are still in a relatively collapsed state, which will change the original physiological curvature of the spine and cause the spinal alignment to shift. Both of these factors affect the structure, mechanical balance, and surrounding tissue status of the spine in different ways, and then affect the occurrence and persistence of RBP [81, 82, 83].

#### 4.3.5 Additional Factors

In addition to the above factors, other factors such as the volume of injected bone cement and different surgical techniques (single or bilateral puncture) may also lead to RBP after PVA. The relationship between the volume of injected bone cement and RBP after PVA involves several key factors. Insufficient bone cement volume is identified as an independent risk factor for postoperative pain, as incomplete filling of vertebral fractures may fail to restore mechanical stability, leading to persistent micro‐movements and neural stimulation. Even with adequate volume, uneven distribution (e.g., unilateral or fragmented placement) can contribute to residual pain, as seen in patients with cement not crossing the vertebral midline demonstrating higher VAS scores compared to those with bilateral symmetric distribution. The relationship between different surgical techniques (unilateral vs. bilateral puncture) and RBP after PVA involves trade‐offs in cement distribution, mechanical stability, and procedural risks. Unilateral puncture offers shorter procedure times, reduced radiation exposure, and lower complication rates but predisposes to asymmetric cement distribution. Bilateral puncture achieves symmetric cement spread, improving stability, whereas increasing operative time, radiation dose, and cement leakage risk.

## 5 Summary

In summary, factors such as thoracolumbar fascia injury, unsatisfactory cement distribution, low pre‐bone mineral density, multiple vertebral fractures, postoperative vertebral body height recovery rate, the volume of injected bone cement, and different surgical techniques (single or bilateral puncture) do not exist in isolation. They influence and interact with each other, jointly disrupting the structural integrity of the spine, interfering with the biomechanical balance, stimulating the surrounding tissues, and affecting nerve conduction from different aspects. Collectively, these factors make RBP after PVA more likely to occur and more difficult to relieve, seriously affecting patients' postoperative quality of life and daily activity abilities. To address the multifactorial risks of RBP after PVA, clinicians should adopt a comprehensive approach. This includes preoperative MRI/CT to evaluate fascial integrity and fracture complexity; bilateral puncture with extrapedicular needle trajectories to optimize cement spread (≥ 50% vertebral coverage) while minimizing fascial trauma; tailored cement volume (1.5–6 mL/side) adjusted for osteoporosis and fracture load; intraoperative fluoroscopic guidance to achieve symmetric cement distribution and restore vertebral height; postoperative core stability training combined with NSAIDs or corticosteroids to reduce fascial inflammation; and close follow‐up for early detection of persistent pain requiring intervention, such as cement revision or targeted physical therapy. By integrating these strategies, mechanical stability is enhanced, fascial function is preserved, and pain pathways are mitigated, improving overall outcomes for patients.

## 6 Limitations

There are some limitations in the current study. Firstly, the included studies in our systematic review and meta‐analysis were based on cohort, case–control, or case series studies, the selection bias of which may affect the estimated results. Secondly, the definition and evaluation time of RBP varied significantly across studies, which may reflect inherent variability in RBP conceptualization. Despite this variability, a summary of existing definitions was synthesized to facilitate cross‐study comparability. Though sensitivity analyzes revealed no substantial alteration in pooled effect estimates, this could still impact our meta‐analysis results. Therefore, standardized criteria for RBP are needed in future research. Thirdly, this study only discusses partial influencing factors for RBP after PVA, whereas other equally important factors—such as vertebral compression ratio, intravertebral vacuum phenomenon, facet joint injury, etc.—remain unaddressed. Clinicians should implement corresponding preventive measures for these factors in clinical practice.

## 7 Conclusions

The current study was conducted to estimate the prevalence and risk factors of RBP after PVA treatment for OVCFs. The results demonstrated that the prevalence of RBP following PVA is significantly high among patients with OVCFs, highlighting the importance of early screening and intervention for RBP in these populations.

Clinically, it is essential to comprehensively consider various risk factors, including thoracolumbar fascia injury, unsatisfactory cement distribution, and low pre‐bone mineral density, to accurately assess patients' conditions and formulate targeted treatment and rehabilitation plans to alleviate RBP.

Furthermore, additional multinational validation studies are required to explore the potential reasons underlying the higher prevalence of RBP after PVA in OVCF patients. Research to improve mechanisms for screening, preventing, and managing RBP after PVA is also needed.

## Author Contributions

**Wenlong Li:** conceptualization, methodology, writing – original draft, funding acquisition. **Bing Zhang:** data curation, formal analysis, visualization. **Chencheng Mei:** investigation, resources, writing – review and editing. **Hui Li:** data curation. **Ruizheng Zhu:** methodology, software, validation. **Hao Lin:** formal analysis, investigation, writing – review and editing. **Jianmin Wen:** supervision, project administration. **Yang Wu:** validation, writing – review and editing. **Xianzhi Ma:** conceptualization, supervision, writing – review and editing.

## Conflicts of Interest

The authors declare no conflicts of interest.
